# Clinical Significance of the Static and Dynamic Q-angle

**DOI:** 10.7759/cureus.24911

**Published:** 2022-05-11

**Authors:** Apostolos Z Skouras, Asimakis K Kanellopoulos, Sophia Stasi, Athanasios Triantafyllou, Panagiotis Koulouvaris, Georgios Papagiannis, George Papathanasiou

**Affiliations:** 1 Sports Excellence, 1st Department of Orthopaedic Surgery, National and Kapodistrian University of Athens School of Medicine, Athens, GRC; 2 Biomechanics and Gait Analysis Laboratory “Sylvia Ioannou”, Orthopaedic Research and Education Center “P.N.Soukakos”, 1st Department of Orthopaedic Surgery, National and Kapodistrian University of Athens School of Medicine, Athens, GRC; 3 Department of Physiotherapy, University of Thessaly, Lamia, GRC; 4 Laboratory of Neuromucsular and Cardiovascular Study of Motion (LANECASM), University of West Attica, Athens, GRC; 5 Department of Physiotherapy, University of West Attica, Athens, GRC; 6 Department of Physiotherapy, University of the Peloponnese, Sparta, GRC

**Keywords:** frontal plane projection angle, dynamic knee valgus, dynamic q-angle, q-angle, knee biomechanics

## Abstract

Q-angle represents the resultant force vector of the quadriceps and patellar tendons acting on the patella. An increased Q-angle has been considered a risk factor for many disorders and injuries. This literature review challenges the clinical value of static Q-angle and recommends a more dynamic movement evaluation for making clinical decisions. Although there are many articles about static Q-angle, few have assessed the value of dynamic Q-angle. We searched Scopus and PubMed (until September 2021) to identify and summarize English-language articles evaluating static and dynamic Q-angle, including articles for dynamic knee valgus (DKV) and frontal plane projection angle. We also used textbooks and articles from references to related articles. Although static Q-angle measurement is used systematically in clinical practice for critical clinical decisions, its interpretation and clinical translation present fundamental and intractable limitations. To date, it is acceptable that mechanisms that cause patellofemoral pain and athletic injuries have a stronger correlation with dynamic loading conditions. Dynamic Q-angle has the following three dynamic elements: frontal plane (hip adduction, knee abduction), transverse plane (hip internal rotation and tibia external rotation), and patella behavior. Measuring one out of three elements (frontal plane) illustrates only one-third of this concept. Static Q-angle lacks biomechanical meaning and utility for dynamic activities. Although DKV is accompanied by hip and tibia rotation, it remains a frontal plane measurement, which provides no information about the transverse plane and patella movement. However, given the acceptable reliability and the better differentiation capability, DKV assessment is recommended in clinical practice.

## Introduction and background

In biomechanics and anthropometry, many methods have been developed to measure lower extremity alignment using various anatomical variables. An anatomical variable, which provides useful information about the knee joint’s alignment and is associated with the femur and tibia’s alignment in the frontal plane, is the quadriceps angle (Q-angle) [1]. According to many studies, the first to use the term “Q-angle” was Brattström in 1964, even though Chen et al. claim the term was used even earlier by Cruveilhier in 1847 [2].

Q-angle is defined as the angle formed between an imaginary line connecting the anterior superior iliac spine (ASIS) of the pelvis to the patella’s midpoint and a proximal projection of the line running from the tibial tubercle to the patella’s center [3-5]. It has been suggested that the Q-angle represents the frontal plane resultant force vector of the quadriceps musculature and patellar tendons acting, respectively, on the patella [6-9]. This definition is typically used for the static Q-angle.

The clinical significance of static Q-angle is under investigation, as an increased Q-angle is considered a risk factor for many disorders or injuries such as patellofemoral pain (PFP), patellar subluxation and dislocation, chondromalacia patellae, knee osteoarthritis, overuse injuries, anterior cruciate ligament injury, patellar instability, disturbances on dynamic balance, and ankle sprains [3,10-18]. Decreased Q-angle may be associated with chondromalacia, patella alta, patellar instability, and PFP [19-21]. The observed inconsistency in the literature regarding the magnitude of correlation of an excessive Q-angle degree with specific pathological manifestation triggers a particular interest in further exploring its value [22-25].

In the past decades, with research progress on lower limb pathomechanics, a new notion was introduced, termed “dynamic Q-angle.” Dynamic Q-angle is defined as the Q-angle through the knee joint’s flexion, with or without a dynamic activity [26-28]. Its measurement requires either the same bony points as on static Q-angle or using dynamic knee valgus (DKV) through the measurement of frontal plane projection angle (FPPA) [29,30]. The DKV is defined as a combination of hip adduction and internal rotation and knee abduction, with the lower limb fixed on the ground [7]. The FPPA is formed by lines connecting the ASIS, the midpoint of the femoral condyles, and the malleoli’s midpoint in the frontal plane [31].

It is acceptable that mechanisms that cause PFP have a stronger correlation with dynamic, not static, loading conditions because of higher muscular and mechanical demands that are needed to perform an activity [32-35]. For this reason, recently, the investigation of the clinical value of lower extremity biomechanics in dynamic activities is constantly gaining ground [36-38].

Q-angle is a human contrivance to clinically interpret the line of pull of the quadriceps and, consequently, explain various syndromes and injuries, as well as better understand human movement. Despite its questionable clinical value, Q-angle has been extensively studied to date. The result is that static Q-angle measurement is used systematically in clinical practice for critical clinical decisions [39]. The values of dynamic Q-angle in clinical practice have similarly been proposed [40,41].

There are serious problems in measuring both static and dynamic Q-angle [42,43]. Therefore, there is no agreement on the optimal way of measuring the angle, both static and dynamic. Serious problems are also observed with the correlation of static and dynamic Q-angle with the lower limb’s biomechanical behavior and its correlation with various clinical manifestations. Therefore, there is no agreement on the clinical value of static and dynamic Q-angle. The purpose of this review is to present data and concerns about the clinical significance of static and dynamic Q-angle.

## Review

An online search of journal databases PubMed and Scopus was performed. The following keywords were used as search terms in various combinations: Q-angle, dynamic Q-angle, dynamic knee valgus (DKV), and frontal plane projection angle (FPPA). Textbooks and articles from references to related articles were selected based on their relevance and specificity.

Range of Q-angle degrees

A review of the literature indicates that the standardized, average degrees of static Q-angle differ between the two genders; in men, it is about 10° to 15°, while in women, it ranges from 15° to 20° [4]. However, there is evidence that Q-angle differs among races as well [44-46]. In particular, women in Nigeria present a range of 20° to 28°, while women in India do not exceed an average of 15° [44,45]. On the other hand, in a study conducted in Arab countries, the average degree for women was 17.35 ± 0.225°, while for men it was 14.1 ± 0.21° [46].

There seems to be a large overlap in the range of Q-angle degrees between asymptomatic and symptomatic individuals. Q-angle degree ranges between 1° and 23° in asymptomatic men, between 0° and 22° in men with unilateral PFP, and between 9° and 22° in men with bilateral PFP [47]. Simultaneously, the Q-angle in asymptomatic women ranges from 2° to 21°, in women with unilateral PFP from 0° to 22°, and in women with bilateral PFP from 2.5° to 30° [47]. Consequently, the difficulty of determining the normal or abnormal Q-angle degrees and finding a cut-off point that could potentially be a predisposing factor for PFP is perceived.

Anatomical factors, such as a wide pelvis, appear to be associated with a higher Q-angle degree [4,5,48]. This is considered the main factor that warrants higher values among women [4,49,50]. However, it has been shown that people of the same height have non-statistically significant differences in Q-angle degrees regardless of gender. The higher the subject, the lower the values, which justifies the difference in reference values between the sexes. In particular, trigonometric analysis has shown that for a 5° difference between two people 168 cm tall, their difference in the distance of the ASIS should differ by 8.6 cm, which is not observed in people of the opposite sex with the same height [46,51,52].

Measuring Q-angle

**Figure 1:**
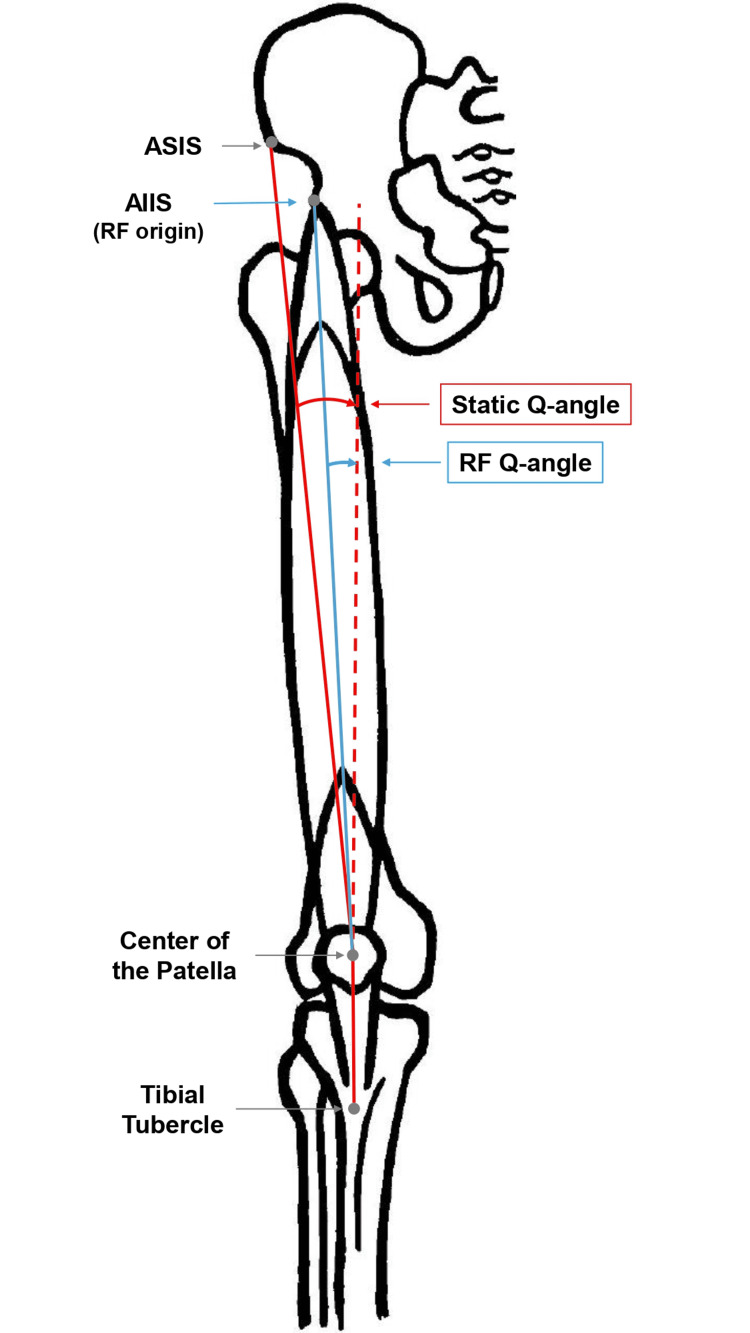
Static Q-angle. Q-angle is defined as the angle formed between an imaginary line connecting the ASIS of the pelvis to the center of the patella and a proximal projection of the line running from the tibial tubercle to the patella’s center (red line). It appears that the measured static Q-angle does not reflect the true line of pull of the RF (blue line), which originates from the AIIS. Figure created by Harris Simeonidis. AIIS: anterior inferior iliac crest; ASIS: anterior superior iliac spine; RF: rectus femoris

The static Q-angle (Figure 1) can be measured in an upright or supine position [3,53,54]. There are two choices regarding knee joint placement in both body positions: (a) fully extended or (b) beyond 20° of flexion [3,54]. The measurement in a supine position, with the knees extended and the quadriceps relaxed, is considered conventional or traditional [54]. Abdel-aziem et al. [55] claim that it is important to measure in an upright position only for those with PFP symptoms, without specifying the position of the knees when measuring in an upright position. A significant and predictable reduction in static Q-angle measured in the unilateral standing position has also been reported in asymptomatic women. In contrast, the same predictable pattern was not observed in symptomatic women with PFP [56]. Several studies have shown differences between the two lower limbs for static Q-angle, although such differences are clinically significant in rare cases [45,46,49,57-60].

**Figure 2:**
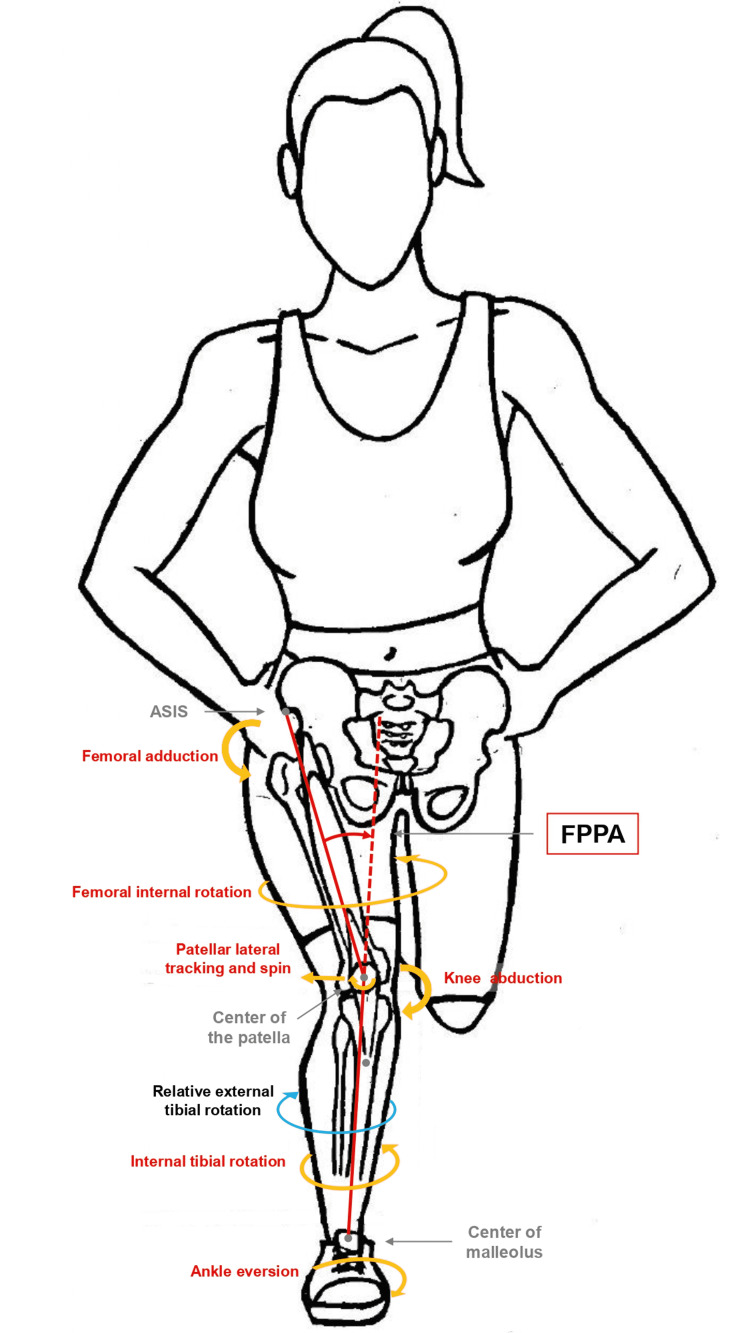
Dynamic Q-angle. Dynamic Q-angle is defined as the Q-angle through the knee joint’s flexion, with or without a dynamic activity. Its measurement requires either the same bony points as on static Q-angle or using DKV through the measurement of FPPA. The DKV is a combination of hip adduction and internal rotation and knee abduction, with the lower limb fixed on the ground. The FPPA is formed by lines connecting the ASIS, the midpoint of the femoral condyles (or the center of the patella), and the malleoli’s midpoint in the frontal plane. Although an ankle eversion normally accompanies tibial internal torsion, a subsequent greater internal rotation of the femur leads to a relative external rotation of the tibia in relation to the femur. This notion cannot be measured directly via FPPA because dynamic Q-angle has three elements: frontal plane (hip adduction, knee abduction), transverse plane (hip internal rotation, tibia external rotation), and patella behavior. Measuring one out of three elements (frontal plane) illustrates only one-third of the concept. Figure created by Harris Simeonidis. ASIS: anterior superior iliac spine; DKV: dynamic knee valgus; FPPA: frontal plane projection angle

Similarly, to measure the dynamic Q-angle (Figure 2), the angle is recorded along the entire motion trajectory, usually in closed kinetic chain (CKC) activities. The most accurate method is three-dimensional (3D) kinematic analysis systems, such as photogrammetry or camera motion capture systems using reflective markers. Alternatively, in two-dimensional (2D) systems, the DKV is recorded at the frontal plane [28,61]. Recently, Llurda-Almuzara et al. [62], using Kinovea as a freely available application for the camera of the phone, reported normative values (mean ± standard deviation) of FPPA as 12.06 ± 7.60° for the right leg and a median ± interquartile range (IQR) of 9.5 ± 13.8° for the left leg. Similar to static Q-angle, they reported inconsistent results compared to previous studies, making it clear that introducing a standardized and valid measurement method remains difficult, as Philp et al. have pointed out [43]. In contrast to static Q-angle, statistically significant differences have been reported between the two lower limbs in activities such as walking and running in the dynamic Q-angle value [63].

The Q-angle value is affected by the hip’s rotational position and the foot’s position. Both the static and dynamic Q-angle amplitude increase significantly when, with the foot fixed to the ground, the hip is brought in from the outside inward and when the subtalar joint comes from supination to pronation [7,64,65]. An additional element that seems to reduce the value of Q-angle significantly is the activation of the quadriceps [50,66]. Although in an upright position, the distance between the ASIS, on both sides, is likely to be greater, the static Q-angle’s value does not vary, regardless of the body’s position [8,66].

The effect of cognitive functions on biomechanics has gained scientific interest recently. In particular, it has been found that in some individuals, during dynamic activities, physical fatigue in combination with perceptual-cognitive tasks increases the maximum abduction of the knee compared to physical fatigue. The latter already appears to have a significant effect on knee mechanics as a variable alone [67,68]. Moreover, similar results have been found in people with visual-spatial memory deficits [69]. Finally, kinesiophobia also plays an important role in the knee’s biomechanics when performing dynamic activities. However, there is currently no data directly linking it to the dynamic Q-angle [70].

Reliability of Static and Dynamic Measurement of Q-angle

Reliability varies depending on the Q-angle measurement technique. Conventional static Q-angle, measured on plain radiograph with a goniometer, shows low-to-moderate reliability, both in adults (intra-rater intraclass correlation (ICC): 0.22-0.75; inter-rater ICC: 0.20-0.70) and in children (intra-rater: 0.42; inter-rater: 0.35) [33,54,71-73]. In contrast, static Q-angle measurement using 2D (MRI or camera) and 3D motion analysis systems using cameras for capturing (system-based measurements) presents excellent intra-rater and inter-rater reliability (ICC: 0.989 and 0.94-0.989, respectively) [33,74,75].

Dynamic Q-angle measurement also shows higher reliability than static Q-angle measurement with a goniometer (conventional measurement) (intra-rater ICC: 0.74-0.998; inter-rater ICC: 0.837-0.913) [76-78]. However, recently, Philp et al. have disputed that the reported DKV values reflect the true knee valgus [43]. They assumed that reported values of DKV greater than 25° are too large to be considered a valid and true measurement, given the possible error from marker movement during dynamic activities. Consequently, they have detected an inconsistency in managing soft-tissue artifacts and proper alignment of the knee axis [43].

Both static Q-angle measurement methods, conventional or system-based, appear to present poor differentiation capability to discriminate/distinguish patients with PFP or asymptomatic individuals [33,74,79]. Conversely, a study reported that Q-angle’s dynamic measurement method has adequate differentiation capability among subjects with and without PFP [33]. Specifically, the aforementioned study’s receiver operating characteristic (ROC) analysis yielded a moderate area under the curve (AUC) value of 0.74 and an acceptable combined sensitivity (67%) and specificity (75%) [33]. These results suggest that dynamic Q-angle may present better differentiation capability than static Q-angle measurement methods [33,61,79,80].

Image-Based Values Compared With Clinically (Static and Dynamic) Measured Q-angle

There is controversy regarding the degree of agreement between static Q-angle measured with a goniometer and its radiological measurement with the help of plain radiographs and CT scans [23,54]. In the systematic review by Smith et al., the three studies included reported poor (ICC: 0.13-0.32) moderate (r = 0.47), and significantly strong (p = 0.05) correlations [51,71,81,82]. In two other studies by Freedman et al. and Draper et al., it appears that the static Q-angle does not reflect the true line of pull of the quadriceps [72,83]. In particular, in the first, the correlation between MRI-based measurement and static Q-angle measured with a goniometer in three different ways (a: the hip and knee fully extended and the quadriceps fully relaxed; b: the hip and knee fully extended and with maximum isometric quadriceps contraction; and c: the knee bent to 15° with the quadriceps relaxed) was weakly to moderately correlated (r = 0.50, 0.48, and 0.58, respectively; p < 0.001) [72]. In the second study, there was a moderate correlation between goniometer measurement (short arm and long arm) and MRI [short arm: r^2^ = 0.44, (p = 0.04); long arm: r^2^ = 0.40 (p = 0.06)] [83]. In contrast, in another recent study, there was a good correlation between three different clinical measurements (upright position with relaxed quadriceps, supine position with relaxed quadriceps, and supine position with contracted quadriceps) and radiological evaluation in osteoarthritis patients with varus knees [r = 0.676 (p < 0.001), r = 0.616 (p < 0.001), r = 0.676 (p < 0.001), respectively] [84].

Simultaneously, concerning one of the three elements of the dynamic Q-angle (frontal plane), in landing activities, there is a good correlation (r = 0.619; p < 0.001) between the 2D and 3D measurements regarding the kinematic of the lower limb in the frontal plane [85,86]. Similarly, in the real-time observational screening of vertical drop jump landing and single-leg squat, physiotherapists demonstrate high reliability to discriminate between subjects with high and low DKV [87,88]. However, physiotherapists’ observation was poorly correlated with knee abduction moments [87]; hence, a qualitative assessment is not recommended, both for experienced and novice clinicians [87,89-91]. Moreover, Di Staulo et al. have found a broad variability in 3D hip and knee kinematics during a single-limb squat in women with PFP, even when they satisfy the visual criteria for a diagnosis of DKV [92].

Discussion

Static Q-angle measurement has been extensively studied as it appears to be correlated with PFP, patellar subluxation and dislocation, chondromalacia patellae, patellofemoral joint osteoarthritis, overuse injuries, anterior cruciate ligament injury, secondary patellar instability, and disturbances on dynamic balance and ankle sprains [3,10-15,17,18,93]. However, this correlation is only found when other pathological variables coexist [58,94-96].

The statically measured Q-angle as a biomechanical factor has five main limitations. First, Q-angle is defined as expressing the direction of pull of the quadriceps muscle’s resultant force, which has not been proven in research [72]. This specific reasoning lacks biomechanical rationale because instead of the resultant force vector of the quadriceps’ four heads, each of which acts on the patella at a different angle of traction, Q-angle’s measurements are based on anatomical protrusions. Consequently, two individuals with the same static Q-angle value cannot imply that they will have the same quadriceps muscle force direction relative to the patella. During a movement, the pattern of muscle activation is not predicted, nor is it the same.

Second, the static Q-angle is constantly changing when performing dynamic activities. Static Q-angle measurement would be more meaningful clinically if it remained stable throughout knee movements or if its fluctuation followed a specific pattern throughout the motion. That would allow dynamic Q-angle to be accurately predicted based on the original/baseline static measurement. Unfortunately, to date, no such thing has been proven [27,30].

In particular, when a person performs a lower limb activity, especially in a CKC, the relationship of ASIS to the center of the patella and the tibia tubercle is constantly changing, and hence, the Q-angle also changes dynamically [97]. The manner and magnitude with which the Q-angle changes to dynamic activities depend on various parameters, such as individual biomechanical characteristics, movement pattern (kinematic and kinetic), and any underline pathology [33,61,98,99]. This fact, combined with the fact that measuring Q-angle in functional activities is not an easy task, is probably the cause of conflicting published results in investigating the relationship of static Q-angle with various pathological entities, especially in the knee. Conversely, studies have been published presenting a correlation between exceeded DKV, acting knee loads, and patellofemoral pathologies, which better explains the role of pathological motor patterns in dynamic situations, particularly in CKC [33,61,100].

Third, because the patella is located within the quadriceps tendon, it does not necessarily follow the femur’s movements, especially during the quadriceps muscle contraction [101]. This may be justified by the fact that 30°-45° knee flexion is considered the most stable position for the patella during isometric contraction of the quadriceps muscle in both OKC and CKC [102]. Thus, the patella’s external dislocation in CKC activities may result from the femur’s excessive rotation below the patella [102]. Therefore, the dynamic study of lower extremity curves should probably be further investigated and correlated with pathologies rather than statically measured Q-angle or patellar movement as a single factor.

A lateral and cranial traction force is exerted on the patella during a dynamic activity due to the quadriceps muscle contraction. It appears that the increased value in static Q-angle may not be an accurate indicator of patella subluxation as it is related to the medial and caudal position of the patella [72,103]. As expected, the increase in the patella’s lateral traction forces, which results in a higher dynamic Q-angle, increases the patellofemoral joint’s outward pressure [7]. The same, however, cannot be claimed for the static Q-angle [104,105].

In addition, there is conflicting evidence regarding the association of lower extremity kinematics and muscle activation of the involved muscles. According to McAllister and Costigan, the kinematic evaluation of dynamic activities alone, such as the double-leg squat, may provide misleading results regarding the symmetry and quality of movement [99]. On the other hand, Malfait et al. [106] argue that there is a clear correlation between the pattern of muscle activation and the kinematics of the lower extremities in drop jump landing. Thus, there is an apparent inability of the static measurement model of Q-angle to satisfactorily reflect the dynamic function of the lower limb, and, consequently, the dynamic Q-angle.

Fourth, concerning normal static Q-angle values, due to racial differences and the overlap of values between symptomatic and asymptomatic individuals, it might be more beneficial to set normal limits on a range of values rather than deviations from the average to be considered as divergent or pathological values [8]. Therefore, determining pathological values, that is, values that will substantially affect the clinician’s clinical reasoning, becomes challenging.

Fifth, even if the aforementioned surrogate outcomes are ignored, static Q-angle does not correlate sufficiently with patient-important outcomes, such as injury incidence, PFP, or anterior cruciate ligament injury, in contrast to DKV [15,22,24,25,107-111].

Despite its weaknesses, the dynamic Q-angle appears to be a promising biomechanical parameter compared to the static Q-angle. It indicates better differentiation capability between people with PFP and asymptomatic counterparts [33,79,80]. Although the DKV has higher intra- and inter-rater reliability [76-78] and normative values have been reported recently [62], DKV measurement includes only one of the three elements of the dynamic Q-angle, that is, the frontal plane of the tibiofemoral axis. Neither the joint’s transverse plane nor the actual position of the patella. Therefore, problems remain to be solved concerning a weighted measurement method and its contribution to the clinician’s clinical reasoning formulation. Finally, further research is required to investigate the agreement between 3D and 2D (frontal plane) measurements and explore their reliability in various activities.

Dynamic Q-angle has three elements, namely, frontal plane (hip adduction, knee abduction), transverse plane (hip internal rotation, tibia external rotation), and patella behavior. Measuring one out of three elements (frontal plane) illustrates only one-third of the concept. Although we know that DKV is accompanied by hip and tibia rotation, it remains a frontal plane measurement, which provides no information about the transverse plane and patella movement. Future studies should consider these variables and use 3D systems to establish normative values between healthy individuals and patients with conditions such as PFP.

## Conclusions

Static Q-angle is a widespread biomechanical parameter among clinicians and researchers. Despite its questionable value, static Q-angle has already been extensively studied, and many clinicians continue to use it systematically for critical clinical decisions. However, its clinical value does not seem to be supported by the current literature, as it presents fundamental and intractable limitations. The lack of reliability and differentiation capability, doubtful representation of the real line of pull of the quadriceps, and the inability to translate static Q-angle into dynamic activities with a defined and predictable movement pattern are some of the limitations. A statically measured angle lacks biomechanical meaning and utility for dynamic activities.

In contrast, DKV (measured at the frontal plane) has high reliability and differentiation capability. Dynamic Q-angle (frontal and transverse plane) seems to be a promising biomechanical parameter but should be investigated more extensively for weighting assessment methods and its clinical value in predicting, preventing, and rehabilitating various painful syndromes and injuries. Due to the expensive equipment needed in the clinical setting and the impractical and challenging way to measure dynamic Q-angle, clinicians are proposed to replace the static Q-angle with DKV using simple and free apps, such as Kinovea, in their clinical routine.
